# Association Between Self-Reported Weekly Physical Activity Duration and Echocardiographic Cardiac Structure and Function in Adults Aged ≥ 45 Years: A Cross-Sectional Study

**DOI:** 10.3390/jcdd13090467

**Published:** 2026-09-16

**Authors:** Piotr Macek, Michał Fułek, Tomasz Harych, Katarzyna Fułek, Krzysztof Kraik, Barbara Dziadkowiec-Macek, Jarosław Domaradzki, Andrzej Wysocki, Paweł Gać, Rafał Poręba, Małgorzata Poręba

**Affiliations:** 1 Department of Environmental Health, Occupational Medicine and Epidemiology, Wroclaw Medical University, 50-345 Wroclaw, Poland; 2 Department of Cardiology, Marciniak Lower Silesian Specialist Hospital, 54-049 Wroclaw, Poland; 3 Department of Diabetology, Hypertension and Internal Diseases, Institute of Internal Diseases, Wroclaw Medical University, 50-556 Wroclaw, Poland; michal.fulek@umw.edu.pl; 4 Dobromed Medical Centre, 57-200 Ząbkowice Śląskie, Poland; 5 Department of Recreation and Tourism, Wroclaw University of Health and Sport Sciences, 51-617 Wroclaw, Poland; tomasz.harych@awf.wroc.pl; 6 Department of Otolaryngology, Wroclaw Medical University, 50-556 Wroclaw, Poland; katarzyna.fulek@umw.edu.pl; 7 Faculty of Medicine, Wroclaw Medical University, 50-345 Wroclaw, Poland; 8 Department of Child and Adolescent Psychiatry, J. Gromkowski Lower Silesian Specialist Hospital, 51-149 Wroclaw, Poland; dziadkowiecbarbara@gmail.com; 9 Department of Biological Principles of Physical Activity, Wroclaw University of Health and Sport Sciences, 51-612 Wroclaw, Poland; jaroslaw.domaradzki@awf.wroc.pl (J.D.); rafal.poreba@awf.wroc.pl (R.P.); malgorzata.poreba@awf.wroc.pl (M.P.); 10 Department of Radiology and Diagnostic Imaging, 4th Military Hospital, 50-981 Wroclaw, Poland; awysocki@4wsk.pl

**Keywords:** physical activity, echocardiography, diastolic function, cardiac remodeling, left ventricular mass, non-linear association

## Abstract

**Background:** The association between habitual physical activity duration and cardiac structure and diastolic function remains incompletely characterized. **Objective:** To evaluate the relationship between self-reported weekly physical activity duration and echocardiographic parameters in adults aged ≥ 45 years. **Methods:** This exploratory cross-sectional study included 84 adults recruited from a cardiology outpatient clinic and classified into three groups according to weekly physical activity duration: <4 h/week (Group A, *n* = 32), 4–12 h/week (Group B, *n* = 28), and >12 h/week (Group C, *n* = 24). All participants underwent transthoracic echocardiography. Multivariable analyses were adjusted for age, sex, body mass index, systolic blood pressure, arterial hypertension, type 2 diabetes mellitus, and smoking. Exploratory natural cubic spline analyses evaluated physical activity as a continuous exposure. **Results:** Participants reporting 4–12 h/week generally showed the most favorable echocardiographic profile. Significant between-group differences after false discovery rate correction were observed for several indices of cardiac remodeling and diastolic function. The proportions of participants classified as having left ventricular diastolic dysfunction according to the original clinical echocardiographic assessment were 37.5%, 32.1%, and 75.0% in Groups A, B, and C, respectively (*p* = 0.004; q = 0.006). After adjustment, Group C had higher odds of being classified as having diastolic dysfunction than Group B (OR = 8.246; 95% CI: 2.118–32.096; *p* = 0.002). Spline analyses demonstrated significant non-linear associations for LVMI, RWT, mean E/e′, and lateral e′ velocity. **Conclusions:** Weekly physical activity duration was associated with cardiac remodeling and diastolic function. Intermediate activity duration was associated with a more favorable echocardiographic profile; however, these exploratory findings do not establish an optimal exercise dose or harmful effects of higher activity.

## 1. Introduction

### 1.1. Aging and Cardiac Remodeling

Advancing age is associated with progressive vascular and myocardial remodeling, including endothelial dysfunction, oxidative stress, chronic low-grade inflammation, increased arterial stiffness, and changes in ventricular geometry. These processes begin before advanced age and contribute to increasing cardiovascular risk across middle and later adulthood [1,2,3,4,5,6,7,8,9,10,11,12].

### 1.2. Aging and Left Ventricular Diastolic Function

Left ventricular diastolic function depends on active myocardial relaxation and passive chamber stiffness. Alterations in calcium handling, titin-based cardiomyocyte stiffness, extracellular-matrix remodeling, and collagen cross-linking contribute to impaired relaxation and increased filling pressures. With aging, left ventricular volumes tend to decrease while wall thickness and concentricity increase, reducing compliance and predisposing to diastolic dysfunction and heart failure with preserved ejection fraction [13,14,15,16,17,18,19,20].

### 1.3. Physical Activity, Exercise Exposure, and Cardiac Adaptation

Physical activity, exercise, and cardiorespiratory fitness are related but distinct constructs [21]. Contemporary recommendations characterize activity exposure using frequency, intensity, duration, and type, whereas self-reported duration alone captures only one dimension [22,23,24]. Regular physical activity is associated with cardiovascular benefit and may favorably influence cardiac function [25,26,27]. At higher training volumes, however, exercise-induced cardiac remodeling depends on exercise modality, intensity, cumulative exposure, age, and baseline cardiovascular status and may overlap with patterns that resemble pathological remodeling [23,28]. These considerations support cautious interpretation of duration-based activity categories and of any non-linear associations derived from them.

### 1.4. Objective

Despite extensive evidence supporting the cardiovascular benefits of physical activity, the relationship between habitual physical activity duration and echocardiographic measures of cardiac structure and function across middle-aged and older adults has not been fully elucidated [28]. In particular, it remains uncertain whether progressively longer durations of self-reported weekly physical activity are associated with proportionally more favorable cardiac morphology and diastolic performance.

Therefore, the primary aim of the present study was to evaluate the association between self-reported weekly physical activity duration and echocardiographic parameters in adults aged ≥ 45 years, with particular emphasis on left ventricular remodeling and diastolic function. Cardiac structural and functional characteristics were compared among three groups defined according to weekly physical activity duration: <4 h/week, 4–12 h/week, and >12 h/week. The initial hypothesis was that greater habitual physical activity duration would be associated with more favorable indices of cardiac structure and diastolic function. Potential non-linear associations observed in the data were subsequently investigated in exploratory continuous-exposure analyses.

## 2. Materials and Methods

### 2.1. General Characteristics of the Study

This cross-sectional observational study was conducted in a cohort of patients recruited from an outpatient cardiology clinic in Wroclaw, Poland, between 1 January 2023 and 31 December 2023. A total of 112 individuals were assessed for eligibility, of whom 84 were ultimately included in the study and the final statistical analyses.

The study should be considered exploratory. No single primary echocardiographic endpoint was prespecified for an endpoint-specific sample-size calculation. Accordingly, multiplicity across echocardiographic outcomes was addressed statistically using false discovery rate correction. A sensitivity analysis based on the final sample size of 84 participants and three comparison groups indicated that the study had 80% power at a two-sided α of 0.05 to detect an overall effect corresponding to approximately Cohen’s f = 0.345.

The study protocol was reviewed and approved by the Bioethics Committee of Wroclaw Medical University (Approval No. KB-210/2023). The study was conducted in accordance with the ethical principles of the Declaration of Helsinki. All participants provided written informed consent prior to enrolment after receiving detailed information regarding the study objectives, procedures, and potential implications.

### 2.2. Eligibility Criteria

**Figure 1:**
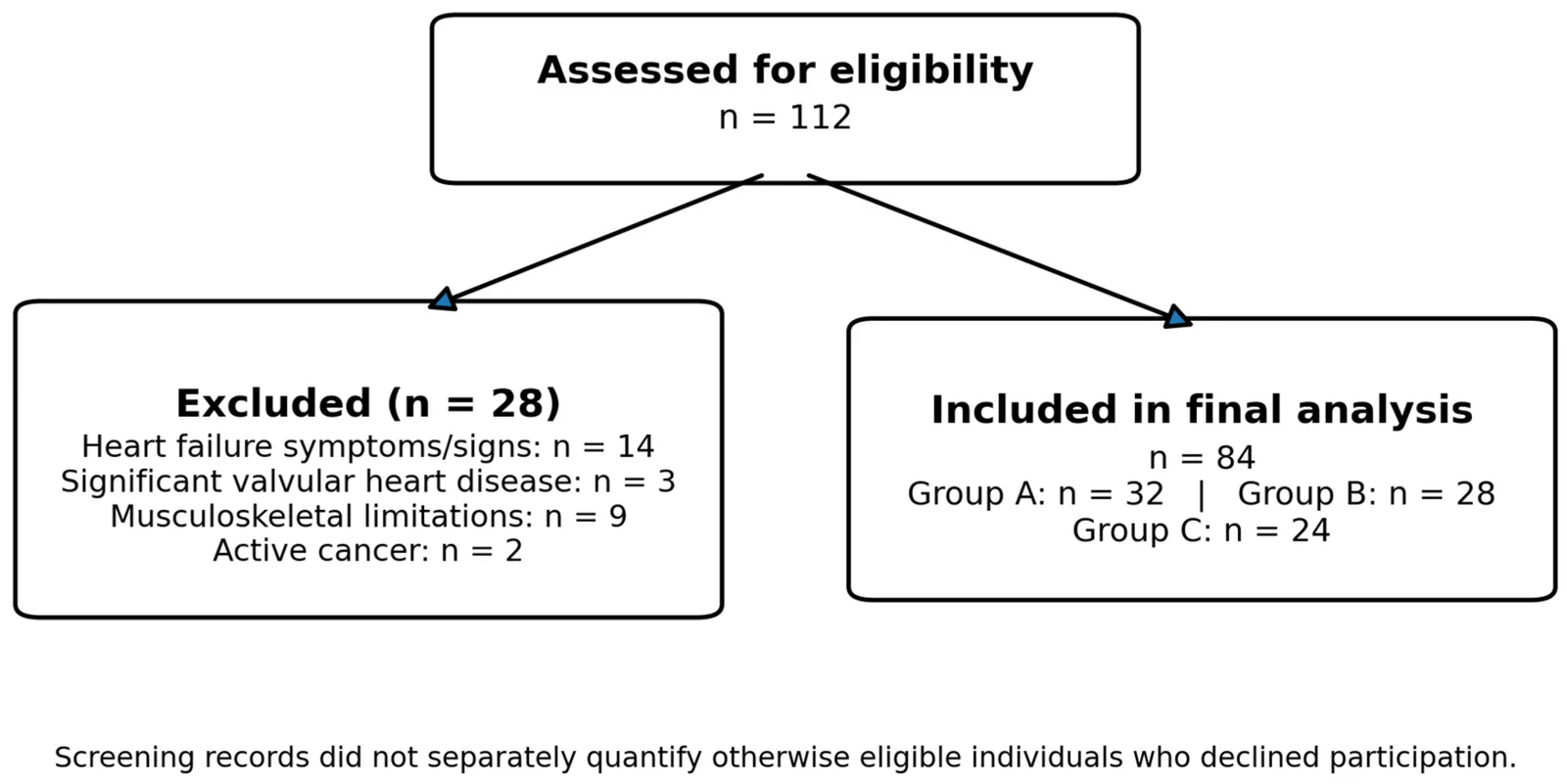
Participant flow through screening and final analysis.

Adults aged ≥ 45 years who were able to comply with the study protocol and provided written informed consent were eligible for enrolment. The ≥45-year threshold was a pragmatic eligibility criterion established for the original cohort to focus recruitment on middle-aged and older adults, in whom age-related cardiac remodeling and diastolic changes were of particular interest. It was not intended as a guideline-defined biological cut-off and was not derived from the present study outcomes. Exclusion criteria included clinically overt cardiovascular, neurological, or psychiatric disorders that could substantially affect the investigated cardiac phenotype or interfere with study procedures. A total of 112 individuals were initially assessed for eligibility. Of these, 28 were excluded because of symptoms or signs of heart failure (*n* = 14), significant valvular heart disease (*n* = 3), musculoskeletal disorders limiting functional assessment (*n* = 9), or active cancer (*n* = 2). Ultimately, 84 participants met the eligibility criteria and were included in the final analysis. The original screening records did not document recruitment as consecutive and did not retain a separate count of otherwise eligible individuals who declined participation; these data therefore could not be reconstructed reliably. Participant flow is summarized in Figure 1.

### 2.3. Physical Activity Assessment

Participants were categorized according to their self-reported average weekly duration of physical activity, assessed using a structured interview administered by trained study personnel. The categories were defined as follows: Group A, <4 h/week; Group B, 4–12 h/week; and Group C, >12 h/week. The assessment included planned exercise, such as walking, cycling, swimming, and gym-based activities, as well as recreational leisure-time physical activity. Participants were asked to report their average weekly physical activity duration during the preceding three months.

The assessment quantified activity duration but did not systematically distinguish activity intensity, frequency, exercise modality, sedentary time, occupational or transportation-related activity, or cardiorespiratory fitness. The structured interview was not a validated physical activity questionnaire. Accordingly, the three categories represent pragmatic self-reported duration groups and should not be interpreted as validated categories of exercise intensity or fitness.

### 2.4. Echocardiographic Assessment

All enrolled participants underwent comprehensive transthoracic echocardiography performed by the same echocardiographer with many years of clinical experience, using a FUJIFILM LISENDO 880LE cardiovascular ultrasound system (FUJIFILM Healthcare Corporation, Tokyo, Japan). Examinations were performed with the system’s adult single-crystal phased-array transthoracic transducer (frequency range 1–5 MHz; sector field of view up to 120°). The assessment included two-dimensional imaging, M-mode echocardiography, color Doppler, pulsed-wave Doppler, continuous-wave Doppler, and tissue Doppler imaging. Standard parasternal, apical, and subcostal views were acquired in accordance with contemporary echocardiographic recommendations. Measurements included left ventricular end-diastolic diameter (LVEDD), left ventricular end-systolic diameter (LVESD), interventricular septal thickness at end-diastole (IVSEDD), posterior wall thickness at end-diastole (PWEDD), right ventricular end-diastolic diameter (RVEDD), left atrial diameter (LA), left atrial volume indexed to body surface area (LAVI), and aortic root diameter. Functional assessment included left ventricular ejection fraction (LVEF) and maximal tricuspid regurgitation velocity (TR Vmax). Tissue Doppler imaging was used to measure early diastolic mitral annular velocities at the septal and lateral annular sites, and the mean E/e′ ratio was calculated.

Left ventricular mass (LVM) was calculated from standard echocardiographic measurements in accordance with contemporary recommendations. Left ventricular hypertrophy was defined as a left ventricular mass index (LVMI) > 115 g/m^2^ in men and >95 g/m^2^ in women.

Participants were classified as having left ventricular diastolic dysfunction (LVDD) according to the original integrated clinical echocardiographic assessment performed during the study period, with reference to the applicable ASE/EACVI recommendations [29]. The clinical assessment incorporated transmitral filling characteristics, septal and lateral mitral annular e′ velocities, the mean E/e′ ratio, LAVI, TR Vmax, and the overall filling pattern. Guideline-based thresholds, including mean E/e′ > 14, septal e′ < 7 cm/s or lateral e′ < 10 cm/s, TR Vmax > 2.8 m/s, and LAVI > 34 mL/m^2^, were considered supportive criteria rather than an automated requirement for a fixed number of positive variables. In the verified analytical dataset, mean E/e′, septal and lateral e′, LAVI, and TR Vmax were available for all participants; the LVDD variable reflected the original clinical echocardiographic classification. The exact software version, a protocol-defined number of cardiac cycles used for averaging, and rhythm-specific averaging procedures were not retained in the analytical study records and could not be reconstructed reliably. Formal blinding to physical-activity information was not prespecified and could not be verified retrospectively. Formal intra- and interobserver reproducibility analyses were not performed.

### 2.5. Clinical and Laboratory Assessment

For all participants enrolled in the study, basic demographic and anthropometric data were collected, including age, sex, and body weight. Body mass index (BMI) was calculated as body weight in kilograms divided by the square of height in meters (kg/m^2^). Blood pressure measurements were performed in accordance with the recommendations of the European Society of Cardiology. After at least 15 min of rest in the seated position, three consecutive blood pressure measurements were obtained [30]. The extreme value was discarded, and the mean of the remaining two measurements was used for further analyses. As part of the diagnostic evaluation, all participants underwent laboratory testing, including measurements of fasting plasma glucose, total cholesterol, and triglyceride concentrations. All analyses were performed using standard enzymatic methods in a certified clinical laboratory. Information regarding cardiovascular risk factors, including cigarette smoking, was obtained from medical records and structured medical interviews. The interview also included an assessment of current pharmacological treatment, with particular emphasis on antihypertensive and lipid-lowering therapies. These data were used to characterize the study population and were considered as potential confounding variables in subsequent statistical analyses.

### 2.6. Statistical Analysis

Statistical analyses of descriptive data and conventional between-group comparisons were performed using Dell Statistica software, version 13.1 (Dell Inc., Round Rock, TX, USA). Additional multivariable and natural cubic spline analyses were performed using Python 3.13 with the statsmodels 0.14.6 and patsy 1.0.2 packages.

Continuous variables are presented as mean ± standard deviation (SD) when parametric assumptions were met and as median [interquartile range (IQR)] when distributions did not satisfy these assumptions. Categorical variables are presented as *n*/N (%). The distribution of continuous variables was assessed using the Shapiro–Wilk test, and homogeneity of variances was evaluated using Levene’s and Brown–Forsythe tests.

To complement significance testing, omnibus effect sizes were calculated for all echocardiographic endpoints. Eta-squared (η^2^) was used for parametric continuous outcomes, epsilon-squared (ε^2^) for outcomes analyzed with the Kruskal–Wallis test, and Cramér’s V for categorical outcomes. Stratified bootstrap 95% confidence intervals were estimated using 2000 resamples. Effect-size estimates are reported in Supplementary Table S1.

Between-group comparisons were performed using one-way analysis of variance (ANOVA) for normally distributed variables with homogeneous variances, Welch ANOVA when the assumption of homogeneity of variance was not satisfied, and the Kruskal–Wallis test for variables not meeting parametric assumptions. Categorical variables were compared using Pearson’s χ^2^ test or an exact test, as appropriate. When an overall comparison was statistically significant, pairwise post hoc comparisons appropriate to the omnibus test were performed. Tukey’s procedure was used following standard ANOVA, whereas Holm adjustment was used for pairwise comparisons following Welch ANOVA, Kruskal–Wallis, or categorical analyses. Exact *p*-values are reported whenever appropriate.

Because multiple correlated echocardiographic outcomes were evaluated, the Benjamini–Hochberg false discovery rate (FDR) procedure was applied across echocardiographic endpoints. Both raw *p*-values and FDR-adjusted q-values are reported in Table 1. An FDR-adjusted q-value < 0.05 was considered statistically significant for interpretation of the multiple echocardiographic comparisons.

To assess whether differences in selected echocardiographic parameters across physical activity-duration categories were independent of clinically relevant potential confounders, multivariable regression analyses were performed. Group B (4–12 h/week) was used as the reference category. Multivariable linear regression models were constructed for LVMI, RWT, LAVI, mean E/e′ ratio, septal e′ velocity, lateral e′ velocity, and TR Vmax. A multivariable logistic regression model was additionally constructed for LVDD. All models used forced-entry adjustment for age, sex, BMI, systolic blood pressure, arterial hypertension, type 2 diabetes mellitus, and smoking. These covariates were selected a priori on clinical grounds and not according to univariate statistical significance. Regression coefficients (Rc) with their standard errors were reported for continuous outcomes, whereas adjusted odds ratios (ORs) with 95% confidence intervals (CIs) were reported for LVDD.

In an exploratory analysis, self-reported weekly physical activity duration was additionally evaluated as a continuous exposure. Potential non-linear associations with selected echocardiographic outcomes were examined using natural cubic spline regression with three degrees of freedom. Spline models were adjusted for the same covariates as the multivariable regression analyses. The overall association between physical activity duration and each continuous outcome was evaluated by comparing the spline model with the corresponding covariate-only model using a partial F-test. Evidence of non-linearity was assessed by comparing the spline model with the corresponding model containing physical activity duration as a single linear term. Adjusted predicted values and 95% confidence intervals were plotted across the observed range of physical activity duration. Because these analyses were exploratory, the spline-derived patterns were interpreted as evidence of potential non-linear associations rather than as estimates of an optimal or harmful physical activity dose.

All statistical tests were two-sided. A *p*-value < 0.05 was considered statistically significant unless FDR-adjusted inference was applied, in which case q < 0.05 was used.

## 3. Results

A total of 84 adults aged ≥ 45 years were included in the study and classified according to self-reported weekly physical activity duration into Group A (<4 h/week; *n* = 32), Group B (4–12 h/week; *n* = 28), and Group C (>12 h/week; *n* = 24). Median weekly physical activity duration was 2.00 [1.00–3.00] h/week in Group A, 8.00 [5.75–9.25] h/week in Group B, and 15.00 [13.75–15.00] h/week in Group C.

The groups did not differ significantly in age, sex distribution, systolic or diastolic blood pressure, prevalence of arterial hypertension or type 2 diabetes mellitus, smoking status, fasting glucose concentration, lipid profile, or the use of antihypertensive, glucose-lowering, and lipid-lowering medications. BMI differed significantly among the groups (*p* = 0.028), with a higher mean BMI in Group B than in Group C in the post hoc comparison (28.87 ± 3.80 vs. 26.05 ± 3.84 kg/m^2^; *p* = 0.039). The remaining pairwise BMI comparisons were not statistically significant. Detailed baseline characteristics are presented in Table 2.

Echocardiographic analysis demonstrated significant between-group differences in multiple measures of cardiac structure and diastolic function. After Benjamini–Hochberg FDR correction, significant differences remained for LVESD, interventricular septal thickness (IVSEDD), posterior wall thickness (PWEDD), left atrial diameter, LAVI, aortic root diameter, LVMI, RWT, mean E/e′ ratio, septal e′ velocity, lateral e′ velocity, TR Vmax, and LVDD.

Participants in Group B generally demonstrated the most favorable structural profile. IVSEDD and PWEDD were lower in Group B than in both Groups A and C. Left atrial diameter was also lower in Group B than in Groups A and C. LAVI was higher in Group C than in both Groups A and B, whereas aortic root diameter and LVMI were significantly higher in Group C than in Group B. RWT differed across all three groups, with the lowest values observed in Group B and the highest in Group C. No significant between-group differences were observed in LVEF.

Measures of diastolic function demonstrated a similar pattern. Mean E/e′ was lowest in Group B and differed significantly between Groups A and B, Groups A and C, and Groups B and C. Both septal and lateral e′ velocities were significantly higher in Group B than in Groups A and C. TR Vmax was significantly higher in Group A than in Group B.

According to the original clinical echocardiographic assessment, 12/32 (37.5%) participants in Group A, 9/28 (32.1%) in Group B, and 18/24 (75.0%) in Group C were classified as having LVDD (*p* = 0.004; FDR q = 0.006). Post hoc comparisons demonstrated a significantly higher proportion of participants classified as having LVDD in Group C than in both Group A (adjusted *p* = 0.014) and Group B (adjusted *p* = 0.008). Although LVH was numerically most frequent in Group C (66.7% vs. 37.5% in Group A and 35.7% in Group B), the overall association did not remain statistically significant after FDR correction (raw *p* = 0.044; q = 0.053). Detailed echocardiographic findings are presented in Table 1.

Omnibus effect sizes with bootstrap 95% confidence intervals are provided in Supplementary Table S1.

Multivariable regression analyses demonstrated that several associations between physical activity-duration category and cardiac structure and diastolic function persisted after adjustment for age, sex, BMI, systolic blood pressure, arterial hypertension, type 2 diabetes mellitus, and smoking.

Compared with Group B, participants in Group C had significantly higher LVMI (Rc = 16.444 ± 4.859; *p* = 0.001), RWT (Rc = 0.149 ± 0.019; *p* < 0.001), LAVI (Rc = 6.067 ± 1.997; *p* = 0.003), and mean E/e′ ratio (Rc = 2.936 ± 0.475; *p* < 0.001), together with significantly lower septal e′ (Rc = −1.583 ± 0.317; *p* < 0.001) and lateral e′ velocities (Rc = −2.459 ± 0.365; *p* < 0.001).

Compared with Group B, participants in Group A also demonstrated higher mean E/e′ (Rc = 1.294 ± 0.438; *p* = 0.004), lower septal e′ (Rc = −1.355 ± 0.293; *p* < 0.001), lower lateral e′ velocity (Rc = −2.230 ± 0.336; *p* < 0.001), higher TR Vmax (Rc = 0.344 ± 0.126; *p* = 0.008), higher LVMI (Rc = 9.291 ± 4.482; *p* = 0.042), and higher RWT (Rc = 0.074 ± 0.017; *p* < 0.001).

In the multivariable logistic regression analysis, participants in Group C had substantially higher odds of being classified as having LVDD than participants in Group B (adjusted OR = 8.246; 95% CI: 2.118–32.096; *p* = 0.002). In contrast, the difference between Groups A and B was not statistically significant (adjusted OR = 1.515; 95% CI: 0.471–4.870; *p* = 0.486). The overall logistic regression model was statistically significant (*p* = 0.025). Detailed results are presented in Table 3.

**Figure 2:**
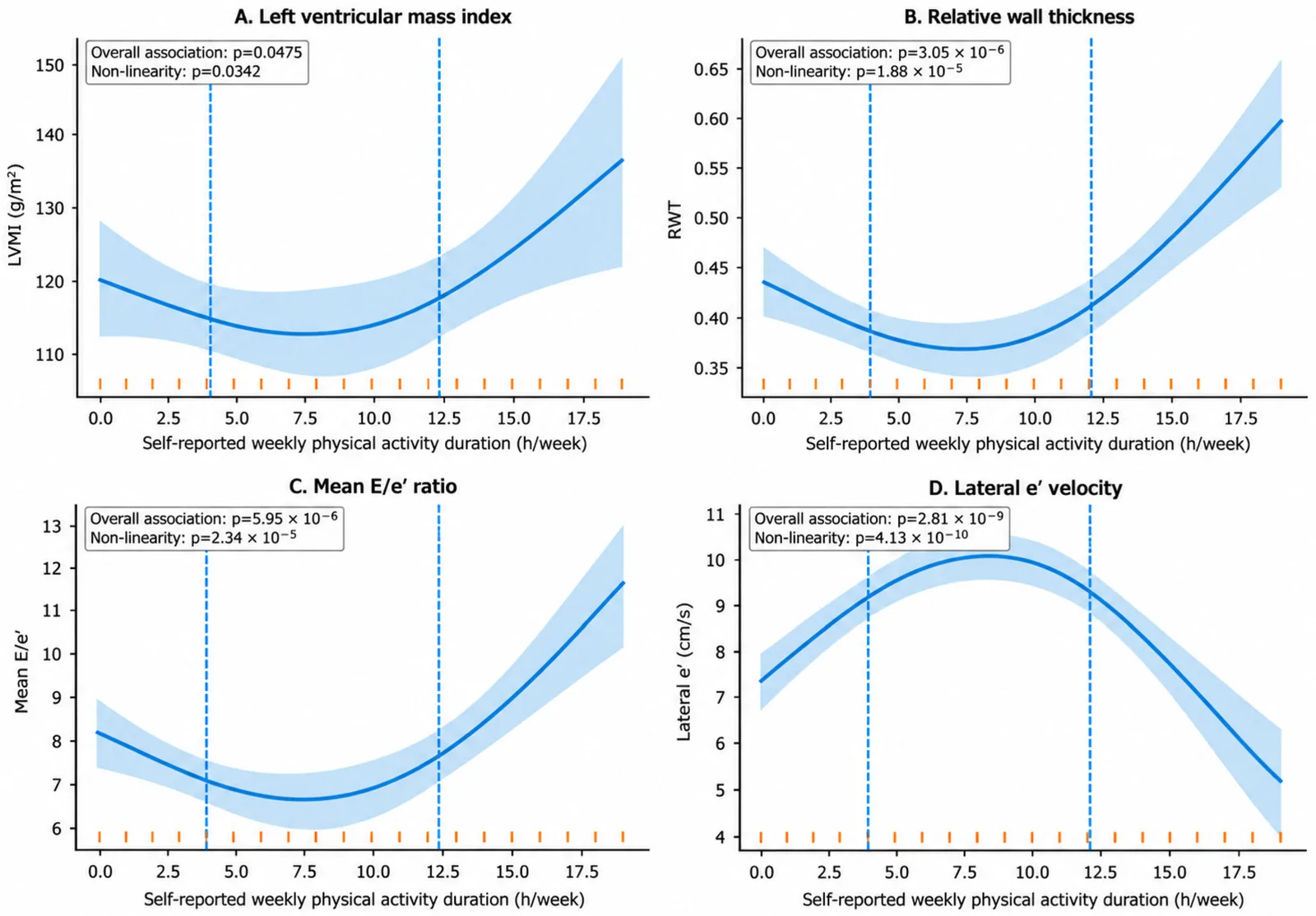
Natural cubic spline analyses of weekly physical activity duration and selected echocardiographic parameters. Adjusted spline curves with 95% confidence intervals for (**A**) left ventricular mass index (LVMI), (**B**) relative wall thickness (RWT), (**C**) mean E/e′ ratio, and (**D**) lateral e′ velocity. Solid blue lines represent adjusted spline estimates, and the blue shaded areas indicate 95% confidence intervals. Vertical dashed lines indicate the reference values (or threshold points) shown by the model. Models were adjusted for age, sex, body mass index, systolic blood pressure, arterial hypertension, type 2 diabetes mellitus, and smoking. Dashed vertical lines indicate the 4 and 12 h/week cut-offs.

Exploratory natural cubic spline analyses provided evidence of non-linear associations between self-reported weekly physical activity duration and several representative echocardiographic parameters. Significant non-linearity was demonstrated for LVMI, RWT, mean E/e′ ratio, and lateral e′ velocity. The fitted curves generally demonstrated lower predicted LVMI, RWT, and mean E/e′ values and higher predicted lateral e′ velocities at intermediate physical activity durations, approximately within the range represented by Group B. For LVMI, the overall association (*p* = 0.0475) and the test for non-linearity (*p* = 0.0342) were statistically significant. Stronger evidence of non-linearity was observed for RWT (overall *p* = 3.05 × 10^−6^; *p* for non-linearity = 1.88 × 10^−5^), mean E/e′ ratio (overall *p* = 5.95 × 10^−6^; *p* for non-linearity = 2.34 × 10^−5^), and lateral e′ velocity (overall *p* = 2.81 × 10^−9^; *p* for non-linearity = 4.13 × 10^−10^). These continuous-exposure analyses support the presence of non-linear associations for selected measures of cardiac remodeling and diastolic function. However, the spline-derived curves should be interpreted as exploratory and do not identify an optimal physical activity dose or establish adverse effects of longer physical activity duration (Figure 2).

## 4. Discussion

### 4.1. Summary of Key Findings

The present study demonstrated an association between self-reported weekly physical activity duration and several echocardiographic measures of cardiac structure and diastolic function in adults aged ≥ 45 years. In the categorical analyses, participants reporting 4–12 h/week of physical activity generally exhibited the most favorable echocardiographic phenotype. This pattern included lower ventricular wall thickness and RWT, lower LVMI, more favorable tissue Doppler indices of myocardial relaxation, and lower estimated filling pressures compared with one or both of the other activity-duration groups.

Importantly, the observed associations were not explained solely by measured differences in baseline cardiovascular risk factors. In multivariable analyses adjusted for age, sex, BMI, systolic blood pressure, arterial hypertension, type 2 diabetes mellitus, and smoking, participants reporting > 12 h/week continued to demonstrate higher LVMI, RWT, LAVI, and mean E/e′ and lower septal and lateral e′ velocities compared with participants reporting 4–12 h/week. The >12 h/week group also demonstrated substantially higher adjusted odds of being classified as having LVDD. Participants reporting <4 h/week similarly showed less favorable values of several diastolic-function parameters compared with the 4–12 h/week group.

The proportion of participants classified as having LVDD was highest among those reporting > 12 h/week and remained significantly different after correction for multiple testing. In contrast, although LVH was numerically more frequent in this group, the corresponding association did not remain statistically significant after FDR correction and should therefore be interpreted cautiously. Left ventricular systolic function, assessed by LVEF, did not differ significantly across the three groups.

The analysis of physical activity duration as a continuous exposure provided additional information beyond the categorical comparisons. Natural cubic spline modeling demonstrated statistically significant non-linear associations for LVMI, RWT, mean E/e′, and lateral e′ velocity, with more favorable predicted values occurring at intermediate durations of self-reported weekly activity. These findings strengthen the evidence that the association may not be strictly linear. Nevertheless, because the spline analysis was exploratory, it should not be interpreted as defining an optimal dose of physical activity or demonstrating that longer activity duration is intrinsically harmful.

### 4.2. Clinical Implications

The present findings should be interpreted primarily as associations between self-reported activity duration and echocardiographic phenotype rather than as evidence supporting a specific exercise prescription. Previous studies have shown that the relationship between physical activity and cardiac structure or function may vary according to age, baseline cardiovascular status, exercise modality, and training history [31,32,33,34,35,36,37]. The present cohort extends these observations to adults aged ≥ 45 years and suggests that simple duration-based categories may conceal potentially non-linear relationships with cardiac remodeling and diastolic function.

At the same time, physical activity duration alone cannot distinguish physiological exercise-related cardiac adaptation from adverse remodeling. Increased wall thickness, left ventricular mass, or atrial dimensions in participants reporting longer activity duration may reflect several possible mechanisms, including differences in exercise type and intensity, previous training exposure, residual cardiovascular risk, or physiological adaptation. Consequently, the present results should not be used to discourage high levels of physical activity or to recommend a specific weekly duration in clinical practice.

### 4.3. Pathophysiological Considerations

Regular physical activity has well-established favorable effects on cardiovascular physiology, including improvements in endothelial function, blood pressure regulation, metabolic profile, inflammatory status, and myocardial performance [38,39]. Conversely, very high volumes of prolonged exercise have been associated in selected populations with distinct structural and physiological adaptations and, under specific circumstances, potential adverse cardiovascular effects [40]. Previous observational studies have also described non-linear associations between physical activity exposure and cardiovascular outcomes [41].

The present findings provide echocardiographic evidence that the relationship between self-reported weekly physical activity duration and selected measures of cardiac structure and diastolic function may likewise be non-linear. This interpretation is supported not only by the categorical group comparisons but also by formal natural cubic spline analyses for LVMI, RWT, mean E/e′, and lateral e′ velocity.

Although left atrioventricular coupling was not assessed in the present study, the observed differences in LAVI may be viewed within the broader concept of atrial–ventricular interaction. Imaging-derived left atrioventricular coupling indices have been proposed as markers of cardiovascular remodeling, and higher CT-derived LACI values have recently been associated with greater cardiovascular risk assessed using the SCORE system [42,43]. However, several alternative explanations should be considered. In participants reporting longer physical activity duration, increased ventricular wall thickness or LVMI may partly represent physiological exercise-related remodeling rather than adverse myocardial damage. Conversely, reverse causality could influence the lower-activity group if participants with less favorable cardiovascular status reduced their habitual activity. Residual confounding related to the intensity, modality, frequency, and lifetime duration of exercise may also contribute to the observed pattern.

Accordingly, the present findings should be interpreted as evidence of a non-linear association rather than a confirmed J-shaped biological dose–response relationship. The data neither establish that 4–12 h/week represents an optimal activity range nor demonstrate that activity exceeding 12 h/week causes adverse cardiac remodeling.

### 4.4. Study Limitations

Several limitations should be considered when interpreting the present findings. First, physical activity was assessed by self-report and the exposure measure was based primarily on weekly duration. The assessment did not systematically quantify exercise intensity, frequency, modality, session duration, sedentary time, occupational or transportation-related activity, or objective cardiorespiratory fitness. The thresholds used to create the three activity-duration categories were therefore pragmatic rather than equivalent to categories derived from a validated physical activity instrument.

Second, physical activity was recalled over the preceding three months, which introduces the possibility of recall bias and may not accurately represent long-term exposure relevant to cardiac remodeling. Seasonal variation in habitual activity could also have influenced the reported duration.

Third, the cross-sectional design precludes conclusions regarding causality or temporal sequence. Reverse causality remains possible because pre-existing cardiovascular or functional characteristics may influence habitual physical activity. The findings therefore cannot establish that differences in physical activity duration caused the observed echocardiographic phenotype.

Fourth, the study was conducted in a relatively small sample recruited from an outpatient cardiology setting. This introduces potential selection bias and limits generalizability to the broader population of adults aged ≥ 45 years. The original screening records did not document recruitment as consecutive and did not retain a separate count of otherwise eligible individuals who declined enrolment. The modest sample size also resulted in limited precision for some multivariable estimates, as illustrated by the wide confidence interval for the association between the >12 h/week category and classification as having LVDD.

Fifth, although the multivariable models adjusted for several clinically relevant cardiovascular risk factors, residual confounding remains possible. Detailed information regarding the duration and long-term control of hypertension, renal function, obstructive sleep apnea, alcohol consumption, functional status, lifetime exercise exposure, and previous athletic training was not available for adjustment.

Sixth, multiple echocardiographic parameters were examined. Although the Benjamini–Hochberg FDR procedure was applied to reduce the probability of false-positive findings, the analyses remain exploratory and should be confirmed in larger independent cohorts.

Seventh, formal intra- and interobserver reproducibility analyses of the echocardiographic measurements were not performed. All examinations were performed by the same echocardiographer with many years of clinical experience using the same FUJIFILM LISENDO 880LE system, which reduced variability related to different operators and ultrasound platforms but does not substitute for quantitative reproducibility assessment. In addition, the exact software version, a protocol-defined number of cardiac cycles used for averaging, rhythm-specific averaging procedures, and formal blinding to physical-activity information were not prospectively documented and could not be reconstructed reliably.

Finally, the continuous natural cubic spline analyses were exploratory and based on a relatively limited number of observations, particularly at the extremes of the physical activity distribution. Although they provided formal evidence of non-linearity for selected outcomes, the position of the predicted minima or maxima should not be interpreted as a precise threshold or optimal exercise dose.

## 5. Conclusions

In this cross-sectional outpatient cohort of adults aged ≥45 years, self-reported weekly physical activity duration was associated with several echocardiographic measures of cardiac remodeling and diastolic function. Participants reporting 4–12 h/week generally demonstrated a more favorable echocardiographic profile than participants in the lower- and higher-duration categories, and several of these associations persisted after adjustment for clinically relevant cardiovascular risk factors.

Exploratory continuous-exposure analyses further demonstrated significant non-linear associations between physical activity duration and selected echocardiographic parameters. These findings are hypothesis-generating and do not establish an optimal weekly duration of physical activity or demonstrate that longer physical activity duration is harmful. Prospective studies using validated and objective measures of physical activity intensity, duration, exercise modality, and cardiorespiratory fitness are required to clarify the shape and clinical significance of these associations.

## Figures and Tables

**Table 1:** Echocardiographic parameters and indices of left ventricular diastolic function according to self-reported weekly physical activity duration.

Parameter	Group A (<4 h/Week; *n* = 32)	Group B (4–12 h/Week; *n* = 28)	Group C (>12 h/Week; *n* = 24)	*p*-Value (FDR q)	Post Hoc
LVEDD (mm)	47.77 ± 7.86	47.41 ± 4.87	50.34 ± 4.49	0.071 (0.080)	–
LVESD (mm)	**33.05 ± 4.44**	**30.01 ± 4.02**	**32.48 ± 4.88**	**0.026 (0.034)**	**A vs. B (*p* = 0.026)**
IVSEDD (mm)	**10.99 ± 1.11**	**9.91 ± 0.83**	**13.66 ± 1.26**	**<0.001 (<0.001)**	**A vs. B (*p* < 0.001);** **A vs. C (*p* < 0.001);** **B vs. C (*p* < 0.001)**
PWEDD (mm)	**10.40 ± 0.52**	**9.08 ± 0.43**	**10.98 ± 1.37**	**<0.001 (<0.001)**	**A vs. B (*p* < 0.001);** **B vs. C (*p* < 0.001)**
RVEDD (mm)	20.73 ± 4.51	21.86 ± 2.81	21.79 ± 2.16	0.462 (0.490)	–
LA (mm)	**42.70 [35.45–48.48]**	**35.90 [34.10–39.38]**	**45.30 [40.27–48.70]**	**<0.001 (0.001)**	**A vs. B (*p* = 0.007);** **B vs. C (*p* < 0.001)**
LAVI (mL/m^2^)	**31.51 ± 6.81**	**31.17 ± 5.43**	**37.08 ± 8.08**	**0.003 (0.006)**	**A vs. C (*p* = 0.009);** **B vs. C (*p* = 0.007)**
Ao (mm)	**33.75 [31.38–37.73]**	**32.90 [31.48–33.90]**	**36.80 [33.72–38.83]**	**0.001 (0.003)**	**B vs. C (*p* < 0.001)**
LVEF (%)	63.85 ± 5.01	63.48 ± 3.74	64.17 ± 7.03	0.890 (0.890)	–
LVMI (g/m^2^)	**117.93 ± 17.25**	**108.39 ± 13.58**	**123.95 ± 19.15**	**0.004 (0.007)**	**B vs. C (*p* = 0.004)**
RWT	**0.40 [0.40–0.50]**	**0.35 [0.30–0.40]**	**0.50 [0.50–0.50]**	**<0.001 (<0.001)**	**A vs. B (*p* = 0.003);** **A vs. C (*p* = 0.002);** **B vs. C (*p* < 0.001)**
LVH, *n*/N (%)	12/32 (37.5%)	10/28 (35.7%)	16/24 (66.7%)	0.044 (0.053)	–
Mean E/E′	**7.65 ± 1.99**	**6.54 ± 1.31**	**9.35 ± 1.40**	**<0.001 (<0.001)**	**A vs. B (*p* = 0.027);** **A vs. C (*p* < 0.001);** **B vs. C (*p* < 0.001)**
Septal E′ (cm/s)	**6.43 ± 1.00**	**7.70 ± 1.01**	**6.20 ± 1.45**	**<0.001 (<0.001)**	**A vs. B (*p* < 0.001);** **B vs. C (*p* < 0.001)**
Lateral E′ (cm/s)	**8.12 ± 1.40**	**10.34 ± 0.96**	**7.85 ± 1.28**	**<0.001 (<0.001)**	**A vs. B (*p* < 0.001);** **B vs. C (*p* < 0.001)**
TR Vmax (m/s)	**2.90 [2.60–3.23]**	**2.60 [2.50–2.60]**	**2.70 [2.38–3.23]**	**0.011 (0.016)**	**A vs. B (*p* = 0.008)**
LVDD, *n*/N (%)	**12/32 (37.5%)**	**9/28 (32.1%)**	**18/24 (75.0%)**	**0.004 (0.006)**	**A vs. C (*p* = 0.014);** **B vs. C (*p* = 0.008)**

Continuous variables are presented as mean ± standard deviation (SD) when parametric assumptions were met; LA, Ao, RWT, and TR Vmax are presented as median [interquartile range (IQR)]. Categorical variables are presented as *n*/N (%). Overall comparisons used one-way ANOVA, Welch ANOVA, or the Kruskal–Wallis test as appropriate; categorical variables were compared using Pearson’s χ^2^ test. Benjamini–Hochberg false discovery rate (FDR) correction was applied across the echocardiographic endpoints; values in the *p*-value column are shown as raw *p*-value (FDR-adjusted q-value). Post hoc pairwise comparisons were performed only after a significant overall test and were adjusted for multiple comparisons using Tukey’s procedure (standard ANOVA) or Holm correction (Welch/Kruskal–Wallis/categorical comparisons). Only statistically significant adjusted pairwise comparisons are shown. Rows in bold remained statistically significant after FDR correction (q < 0.05). Abbreviations: Ao, aortic root diameter; FDR, false discovery rate; IQR, interquartile range; IVSEDD, interventricular septal thickness at end-diastole; LA, left atrial diameter; LAVI, left atrial volume index; LVDD, left ventricular diastolic dysfunction; LVEDD, left ventricular end-diastolic diameter; LVEF, left ventricular ejection fraction; LVESD, left ventricular end-systolic diameter; LVH, left ventricular hypertrophy; LVMI, left ventricular mass index; PWEDD, posterior wall thickness at end-diastole; RVEDD, right ventricular end-diastolic diameter; RWT, relative wall thickness; SD, standard deviation; TR Vmax, maximal tricuspid regurgitation velocity.

**Table 2:** Baseline demographic, clinical, biochemical, and pharmacological characteristics of the study population according to self-reported weekly physical activity duration.

Characteristic	Group A (<4 h/Week; *n* = 32)	Group B (4–12 h/Week; *n* = 28)	Group C (>12 h/Week; *n* = 24)	*p*-Value
Physical activity duration, h/week, median [IQR] (range)	2.00 [1.00–3.00] (0–3)	8.00 [5.75–9.25] (4–12)	15.00 [13.75–15.00] (13–19)	—
Age, years	64.42 ± 9.97	64.56 ± 7.75	66.14 ± 8.87	0.746
BMI, kg/m^2^	26.55 ± 4.43	28.87 ± 3.80	26.05 ± 3.84	0.028
Sex: men, *n*/N (%)	14/32 (43.8%)	13/28 (46.4%)	11/24 (45.8%)	0.976
Sex: women, *n*/N (%)	18/32 (56.2%)	15/28 (53.6%)	13/24 (54.2%)	
Arterial hypertension, *n*/N (%)	13/32 (40.6%)	11/28 (39.3%)	10/24 (41.7%)	0.985
Systolic blood pressure, mmHg	127.58 ± 10.96	126.92 ± 13.73	131.20 ± 12.21	0.411
Diastolic blood pressure, mmHg	81.39 ± 7.14	78.38 ± 7.66	80.13 ± 8.20	0.318
**Antihypertensive medications**				
ACE inhibitors, *n*/N (%)	8/32 (25.0%)	6/28 (21.4%)	4/24 (16.7%)	0.754
β-blockers, *n*/N (%)	7/32 (21.9%)	5/28 (17.9%)	3/24 (12.5%)	0.675
Diuretics, *n*/N (%)	4/32 (12.5%)	3/28 (10.7%)	3/24 (12.5%)	1.000
Calcium channel blockers, *n*/N (%)	4/32 (12.5%)	2/28 (7.1%)	2/24 (8.3%)	0.807
Angiotensin receptor blockers, *n*/N (%)	2/32 (6.2%)	1/28 (3.6%)	1/24 (4.2%)	1.000
Type 2 diabetes mellitus, *n*/N (%)	4/32 (12.5%)	3/28 (10.7%)	3/24 (12.5%)	1.000
Fasting glucose, mg/dL	106.73 ± 16.10	99.38 ± 13.63	102.08 ± 11.78	0.132
**Glucose-lowering therapy**				
Oral glucose-lowering medications, *n*/N (%)	4/32 (12.5%)	3/28 (10.7%)	3/24 (12.5%)	1.000
Insulin therapy, *n*/N (%)	0/32 (0.0%)	0/28 (0.0%)	0/24 (0.0%)	—
Total cholesterol, mg/dL	181.11 ± 43.98	196.24 ± 42.70	177.46 ± 47.44	0.264
Triglycerides, mg/dL	125.22 ± 44.19	127.15 ± 47.80	125.02 ± 46.13	0.982
**Lipid-lowering medications**				
Statins, *n*/N (%)	11/32 (34.4%)	9/28 (32.1%)	8/24 (33.3%)	0.983
Fibrates, *n*/N (%)	4/32 (12.5%)	3/28 (10.7%)	3/24 (12.5%)	1.000
Smoking, *n*/N (%)	5/32 (15.6%)	3/28 (10.7%)	3/24 (12.5%)	0.918

Data are presented as mean ± standard deviation (SD), except physical activity duration, which is presented as median [interquartile range (IQR)] (range). Categorical variables are presented as *n*/N (%). Exact *p*-values are reported. No *p*-value is provided for physical activity duration because this variable defined the study groups. For BMI, the overall between-group difference was significant (*p* = 0.028); Tukey post hoc comparisons: Group B vs. Group C, *p* = 0.039; Group A vs. Group B, *p* = 0.077; Group A vs. Group C, *p* = 0.894. Abbreviations: ACE, angiotensin-converting enzyme; BMI, body mass index; IQR, interquartile range; SD, standard deviation.

**Table 3:** Results of multivariable regression analyses evaluating the association between self-reported weekly physical activity categories and selected echocardiographic markers of cardiac remodeling and diastolic function in adults aged ≥ 45 years (*n* = 84).

**A. Multivariable linear regression analysis.**													
**Dependent variable**	**Group A (<4 h/week) vs. Group B (4–12 h/week)**					**Group C (>12 h/week) vs. Group B (4–12 h/week)**					**Adjusted R^2^**		***p*** **of model**
	**Rc**	**SEM of Rc**		***p***		**Rc**	**SEM of Rc**		***p***				
LVMI (g/m^2^)	9.291	4.482		**0.042**		16.444	4.859		**0.001**		0.102		**0.045**
RWT	0.074	0.017		**<0.001**		0.149	0.019		**<0.001**		0.437		**<0.001**
LAVI (mL/m^2^)	0.804	1.842		0.664		6.067	1.997		**0.003**		0.087		0.068
Mean E/e′	1.294	0.438		**0.004**		2.936	0.475		**<0.001**		0.297		**<0.001**
Septal e′ (cm/s)	−1.355	0.293		**<0.001**		−1.583	0.317		**<0.001**		0.304		**<0.001**
Lateral e′ (cm/s)	−2.230	0.336		**<0.001**		−2.459	0.365		**<0.001**		0.420		**<0.001**
TR Vmax (m/s)	0.344	0.126		**0.008**		0.119	0.136		0.384		0.103		**0.045**
**B. Logistic regression analysis for the dependent variable LVDD.**													
Comparison	**Rc**		**SEM of Rc**		***p***			**Odds ratio**		**95% CI**		***p*** **of model**	
Group A vs. Group B	0.415		0.596		0.486			1.515		0.471–4.870		**0.025**	
Group C vs. Group B	2.110		0.693		**0.002**			8.246		2.118–32.096			

Group B (4–12 h/week) was the reference category in all models. Rc values in panel A represent adjusted mean differences relative to Group B; positive Rc values indicate higher values and negative Rc values indicate lower values compared with Group B. Each model used forced-entry adjustment for age, sex, body mass index, systolic blood pressure, arterial hypertension, type 2 diabetes mellitus, and smoking. This covariate set was selected a priori on clinical grounds and was not based on univariate statistical significance. Panel A presents selected non-redundant markers of cardiac remodeling and diastolic function. Standard errors are reported as SEM of Rc to retain the regression-table format used in prior publications by the authors. Panel B reports adjusted odds ratios with 95% confidence intervals. Abbreviations: CI, confidence interval; LAVI, left atrial volume index; LVDD, left ventricular diastolic dysfunction; LVMI, left ventricular mass index; Rc, regression coefficient; RWT, relative wall thickness; SEM of Rc, standard error of the regression coefficient; TR Vmax, maximal tricuspid regurgitation velocity.

## Data Availability

The data are available from the authors upon reasonable request.

## Supplementary Materials

The following supporting information can be downloaded at: https://www.mdpi.com/article/10.3390/jcdd13090467/s1, Table S1: Omnibus effect sizes for between-group echocardiographic comparisons.
