# Supplementary material for: Association Between Self-Reported Weekly Physical Activity Duration and Echocardiographic Cardiac Structure and Function in Adults Aged ≥ 45 Years: A Cross-Sectional Study
Source: J Cardiovasc Dev Dis. 2026 Sep 16;13(9):467. doi: 10.3390/jcdd13090467 (PMC13607980; doi:10.3390/jcdd13090467)
Supplement: Supplementary file 1 [file jcdd-13-00467-s001.zip › jcdd-4548353-supplementary.pdf]

Supplementary Table S1. Omnibus effect sizes for between-group echocardiographic comparisons.

**Outcome**	**Effect-size metric**	**Estimate (bootstrap 95% CI)**
LVEDD (mm)	η^2	0.042 (0.006–0.164)
LVESD (mm)	η^2	0.086 (0.019–0.229)
IVSEDD (mm)	η^2	0.671 (0.564–0.781)
PWEDD (mm)	η^2	0.470 (0.336–0.646)
RVEDD (mm)	η^2	0.025 (0.001–0.153)
LA (mm)	ε^2	0.160 (0.055–0.329)
LAVI (mL/m^2)	η^2	0.131 (0.034–0.296)
Ao (mm)	ε^2	0.137 (0.041–0.293)
LVEF (%)	η^2	0.003 (0.001–0.107)
LVMI (g/m^2)	η^2	0.125 (0.037–0.275)
RWT	ε^2	0.427 (0.291–0.587)
Mean E/e′	η^2	0.323 (0.212–0.477)
Septal e′ (cm/s)	η^2	0.251 (0.130–0.425)
Lateral e′ (cm/s)	η^2	0.456 (0.350–0.580)
TR Vmax (m/s)	ε^2	0.086 (0.006–0.250)
LVH	Cramér's V	0.273 (0.092–0.483)
LVDD	Cramér's V	0.365 (0.197–0.560)

Effect sizes are descriptive omnibus measures: η^2 for parametric continuous outcomes, ε^2 for Kruskal–Wallis outcomes, and Cramér’s V for categorical outcomes. Confidence intervals were estimated by stratified bootstrap with 2000 resamples.
